# Use of N-Methylmorpholine N-oxide (NMMO) pretreatment to enhance the bioconversion of lignocellulosic residues to methane

**DOI:** 10.1007/s13399-022-03173-x

**Published:** 2022-08-17

**Authors:** A. Oliva, L. C. Tan, S. Papirio, G. Esposito, P. N. L. Lens

**Affiliations:** 1 National University of Ireland Galway, Department of Microbiology and Ryan Institute, University Road, Galway, H91 TK33 Ireland; 2 University of Naples Federico II, Department of Civil, Architectural and Environmental Engineering, Via Claudio 21, 80125 Naples, Italy

**Keywords:** NMMO pretreatment, Anaerobic digestion, Bioenergy, Almond shell, Spent coffee grounds, Hazelnut skin

## Abstract

Lignocellulosic residues (LRs) are one of the most abundant wastes produced worldwide. Nevertheless, unlocking the full energy potential from LRs for biofuel production is limited by their complex structure. This study investigated the effect of N-methylmorpholine N-oxide (NMMO) pretreatment on almond shell (AS), spent coffee grounds (SCG), and hazelnut skin (HS) to improve their bioconversion to methane. The pretreatment was performed using a 73% NMMO solution heated at 120 °C for 1, 3, and 5 h. The baseline methane productions achieved from raw AS, SCG, and HS were 54.7 (± 5.3), 337.4 (± 16.5), and 265.4 (± 10.4) mL CH_4_/g VS, respectively. The NMMO pretreatment enhanced the methane potential of AS up to 58%, although no changes in chemical composition and external surface were observed after pretreatment. Opposite to this, pretreated SCG showed increased porosity (up to 63%) and a higher sugar percentage (up to 27%) after pretreatment despite failing to increase methane production. All pretreatment conditions were effective on HS, achieving the highest methane production of 400.4 (± 9.5) mL CH_4_/g VS after 5 h pretreatment. The enhanced methane production was due to the increased sugar percentage (up to 112%), lignin removal (up to 29%), and loss of inhibitory compounds during the pretreatment. An energy assessment revealed that the NMMO pretreatment is an attractive technology to be implemented on an industrial scale for energy recovery from HS residues.

**Supplementary Information:**

The online version contains supplementary material available at 10.1007/s13399-022-03173-x.

## Introduction

Anaerobic digestion (AD) is one of the most employed and successful strategies for biofuel production [1]. The gaseous output of AD is biogas, a gas mixture mainly composed of carbon dioxide and methane that can be used for several applications depending on the purity and volume [2]. The biogas produced has the advantage of being re-used on-site to maintain the digester temperature, as well as to ensure the energy self-sufficiency of the entire AD plant [3].

Several substrates are employed for AD, including lignocellulosic residues (LRs). LRs mainly originate from farming crops, land management, agricultural and municipal activities, but also confectionery industry and commercial activities, such as bars and cafés [4–6]. These biomass types generate disposal and management issues, impacting rural and urban areas [7, 8]. Being among the most abundant wastes worldwide [9] and due to their low supply cost [10], LRs are highly favourable for bioenergy generation, with an estimated energy potential of 30 EJ per year [11]. However, the complex LR structure, mainly composed of cellulose, hemicellulose, and lignin [12], makes them often ill-suited for AD. For this reason, pretreatments are frequently employed to enhance the hydrolysis of cellulose and hemicellulose sugars, allowing a more profitable AD [13].

Among several LRs, nut residues are attracting the attention of many researchers due to their huge output and potential for biofuel production [14]. Global tree nut production has steadily increased in the last decade, reaching over 5.3 million metric tons in the harvesting season 2020/2021 [15]. The top producing countries are the USA, Turkey, and China, but nuts are exported all over the world, both shelled and unshelled. In particular, European countries cover over 30% of the global nuts consumption. The tree nut supply value rises year by year, reaching a value of 38.8 billion dollars in the 2020/2021 season [15]. However, the tree nut network also generates millions of tons of residues, causing environmental and disposal problems [16]. Indeed, most of the nut residues are nowadays still landfilled or incinerated [16], losing significant amounts of high organic content to be alternatively valorised [17]. Apart from nuts, the coffee production chain is also attractive for residue valorisation via AD [18]. In particular, spent coffee grounds, representing the final waste produced during coffee production/consumption, is an opportunity for AD, with over 6 billion tons of wastes produced every year [18].

This study aims to investigate (i) the AD process and methane potential of three raw LRs, i.e. almond shell (AS), spent coffee grounds (SCG), and hazelnut skin (HS) and (ii) the effect of N-Methylmorpholine N-oxide (NMMO) pretreatment on the LRs looking at the impact on both chemical composition and methane potential. NMMO is an organic solvent able to modify the cellulosic part of the biomass after being mixed with the LRs and heated at 90–130 °C [19]. The effect on cellulose depends on the NMMO concentration, with cellulose fibres swelling up by creating balloons when increasing the NMMO concentration [20]. The presence of swelled fibres enhances the biomass porosity, which is one of the most relevant factors for efficient anaerobic digestion, being an index of the accessible surface area for microbial attack [21]. When using 79% NMMO purity, the cellulose dissolution inside balloons occurs until the balloons break out, releasing the dissolved cellulose when the NMMO concentration exceeds 85% [22]. The dissolved cellulose can be regenerated by adding boiling water as an anti-solvent, obtaining a cellulose-rich material, with lower crystallinity and higher porosity [23]. While 85% NMMO pretreatment leads to a lower degree of cellulose crystallinity, swelling (73%) and ballooning (79%) modes are more efficient in increasing the porosity of the cellulose [20]. On the other hand, pretreatments with NMMO at concentrations lower than 70% are less effective on the cellulose swelling [22]. Therefore, the NMMO pretreatment can enhance the biodegradability of LRs at relatively mild operating temperatures. Although being an expensive reagent, NMMO has the advantage of being environmentally friendly and efficiently recoverable (up to 99%) [19]. Furthermore, the NMMO treatment is already a well-known process on an industrial scale, being used worldwide for the Lyocell process in the textile industry [24].

A few studies investigated NMMO pretreatment to enhance the biodegradability of LRs, mainly focusing on straws and forest residues. However, the growing demand for alternative sources of (bio)energy triggers the exploration of untapped organic substrates, such as nut and coffee residues. Contrary to the most studied LRs, these substrates show higher lignin content and richness in non-structural compounds. The difference in chemical composition can result in different AD performance and effectiveness of pretreatment. In addition, most previous studies investigated the effect of NMMO at 85% concentration [25–27]. However, a lower NMMO concentration not only allows a greater swelling of the cellulose fibres but can also reduce the overall costs of the pretreatment by decreasing the NMMO amount required for the process. Therefore, the present study focused on investigating the efficiency of a low-NMMO concentration (i.e. 73%) pretreatment on the methane production from AS, SCG, and HS compared to the baseline performance, varying the pretreatment time from 1 to 3 and 5 h. The correlation between the biochemical methane potential (BMP), chemical composition, and physical characteristics of the substrate was discussed. The kinetics of the AD process were studied by fitting the experimental data with a modified Gompertz model. An energy gain assessment was carried out to validate the viability of the NMMO pretreatment on a larger scale. Furthermore, economic, energetic and environmental aspects are discussed in the perspective of implementing the NMMO technology on an industrial scale.

## Materials and methods

### Substrate and inoculum

Three LRs were used as substrates for AD, i.e. AS, SCG, and HS. The AS were obtained from shelled almonds purchased in a local grocery store (Lazio Region, Italy). The SCG were collected from a coffee bar (Galway County, Ireland) and dried at 50 °C to avoid spoilage during storage. The HS were supplied by a local food farming company (Campania Region, Italy). HS and AS were cut down and sieved to select a particle size between 1 and 2.5 mm. The three substrates were stored in plastic bags at 4 °C prior to being pretreated or directly used in the AD experiments. The inoculum used as a source of microorganisms was a digestate from buffalo manure (DBM) obtained from a full-scale AD plant located in Eboli (Italy). The characterisation of the raw LRs in terms of total (TS) and volatile solid (VS) and total carbon content is reported in *Table **1*. The DBM was characterised in detail in previous studies, where the same inoculum was used [28, 29].

**Table 1:** Total (TS) and volatile (VS) solid, and total carbon content of raw substrates, i.e. almond shell (AS), spent coffee grounds (SCG), hazelnut skin (HS), and TS and VS of the inoculum, i.e. digestate from buffalo manure (DBM)

	AS	SCG	HS	DBM
TS ^a^ (%)	89.6 ± 0.1	87.4 ± 0.6	89.2 ± 0.3	4.8 ± 0.1
VS ^a^ (%)	88.1 ± 0.3	85.8 ± 0.5	86.6 ± 0.3	2.9 ± 0.0
VS/TS (g/g)	0.98	0.98	0.97	0.60
Total Carbon (%)	50.2 ± 0.1	54.2 ± 0.1	58.1 ± 0.0	-

^a^ TS and VS are based on g/100 g wet matter

^b^ Total carbon content is based on g/100 g TS

### N-Methylmorpholine N-oxide pretreatment

The NMMO pretreatment was performed by mixing 30 g of each substrate with 300 g of 73% (*w/w*) NMMO solution in 1000-mL Erlenmeyer flasks, keeping a substrate-to-solvent ratio of 1:10 (*w/w*) [27]. The 73% NMMO solution was obtained by concentrating the commercial 50% (*w/w*) NMMO (Sigma-Aldrich, St. Louis, USA) using a R210/R215 rotary evaporator (Büchi, Flawil, Switzerland). The flasks containing the mixture LRs-NMMO were heated and kept at 120 °C for 1, 3, and 5 h using an ONE22 oil bath (Memmert, Schwabach, Germany). Before heating the mixture, 0.625 g propyl gallate (ACROS organics, Dublin, Ireland) per kg NMMO solution was added to avert the oxidation of NMMO during the pretreatment [27]. The mixing was done manually every 10 min using a glass stirring rod. After the pretreatment, boiling deionised water was added as an anti-solvent to break the reaction [19]. The solid residues were placed in a textile cloth and washed with abundant boiling deionised water till a clear filtrate was obtained. The pretreated LRs were dried at 50 °C before undergoing AD.

### BMP tests and calculation of biogas production

BMP batch tests were performed under mesophilic (37 ± 1 °C) conditions in 250-mL serum glass bottles (OCHS, Bovenden, Germany). Each bottle was loaded with 1.5 g VS from DBM and 1 g VS from raw or pretreated AS, SCG, or HS. Demineralised water was added to adjust the final working volume to 150 mL, leaving 100 mL as headspace volume for the biogas accumulation. The final solids content of the AD process was 2.3% TS. Control biochemical tests were simultaneously carried out to evaluate the methane production obtained from the inoculum only. Each bottle was flushed for 2 min with argon gas (flow rate of 5 L/min) to ensure anaerobic conditions and then left at atmospheric pressure. All the experiments were performed in triplicate, and the bottles were shaken manually once per day.

The biogas production was quantified by measuring the pressure difference of the headspace volume between two sampling points using a Leo 1 pressure reader (Keller, Winterthur, Switzerland). The pressure value was then converted into volume following the ideal gas law [30]. The carbon dioxide and methane content were evaluated through an Einhorn’s saccharometer (Glass Studio, Naples, Italy), filled with 12% NaOH solution [31, 32] and thymolphthalein as pH indicator (Sigma-Aldrich, St. Louis, USA). The Einhorn’s saccharometer is a glass tool that, based on the principles of the water displacement method, allows measuring the carbon dioxide content in a known volume of gaseous sample. The net cumulative methane production achieved from the AD of raw and NMMO-pretreated LRs was calculated as the average of the biological triplicates after subtracting the methane production of the controls. The methane production was recorded regularly until the daily accumulation in all bottles was below 1% of the cumulative methane production [33].

### Analytical methods

TS and VS of raw and pretreated LRs as well as of the inoculum and the final digestate were determined as described by Sluiter et al. [34, 35], using a TCN115 convection oven (Argo Lab, Carpi, Italy) and a BWF 11/13 muffle furnace (Carbolite, Sheffield, UK), respectively. VS degradation during AD was estimated by comparing the initial and final VS content measured for each bottle. The total carbon content of raw LRs was measured by Celignis Limited (Limerick, Ireland) using a Vario MACRO cube elemental analyser (Elementar, Langenselbold, Germany) following the European Standard EN 15,104:2011 procedure.

The water retention capacity (WRC), an indicator of the accessible interior surface area, of raw and pretreated LRs was measured as suggested by Sanchez et al. [36]. The external surface area of raw and pretreated LRs was observed through scanning electron microscopic (SEM) images, using the procedure and the equipment previously described by Oliva et al. [37]. The untreated and NMMO-pretreated LRs were analysed with a Nicolet iS5 Fourier-transform infrared (FTIR) spectrometer (Thermo Fisher Scientific, Waltham, USA) to evaluate the crystalline structure of the cellulose by determining the lateral order index (LOI) of the samples. LOI was obtained as the ratio between the absorbance at 1420 cm^−1^, representative of the crystalline fraction of the cellulose, and the absorbance at 898 cm^−1^, representative of the amorphous cellulose [38]. The analysis was done in triplicate, and the data were averaged over 16 runs with a resolution of 4 cm^−1^ in the 4000–400 cm^−1^ region.

The characterisation of raw and pretreated LRs in terms of extractives, structural carbohydrates, total lignin and ashes was performed by Celignis Limited (Limerick, Ireland) following the protocols of Sluiter et al. [39, 40]. Firstly, the extractives were removed with a sequential extraction using water and 95% ethanol solution as solvents. Afterwards, the extractives-free LRs underwent a two-step acid hydrolysis using 72 and 4% (*w/w*) H_2_SO_4_ at 30 and 121 °C, respectively. Liquor and acid-insoluble residues were separated by filtration. The acid-soluble lignin was determined spectrophotometrically at 205 nm using a HP 8452A ultraviolet–visible spectroscopy device (Hewlett-Packard, Palo Alto, USA). The Klason lignin was estimated gravimetrically by subtracting the acid-insoluble ash from the acid-insoluble residues. The speciation of the structural carbohydrates solubilised in the two-step hydrolysis was obtained with an ICS-3000 Ion Chromatography System (DIonex, Sunnyvale, USA).

Volatile fatty acids (VFAs) accumulation and degradation during the AD process were monitored by sampling 1.5 mL of the liquid phase from each bottle seven times during the first 14 days of the experiment. The samples were stored and prepared for analysis as described by Papirio [29]. The method and equipment used for VFAs analysis are reported by Bianco et al. [41]. The pH of the liquid samples was measured using a HI-98103 pH meter (Hanna Instruments, Woonsocket, USA).

### Kinetic model

The kinetics of methane production obtained from raw and pretreated HS, SCG, and AS were evaluated by fitting the experimental data with a modified Gompertz model [42], using Eq. (1):

1 $$G(t)=G_m\cdot\exp\left\{-exp\left[\frac{R_m\cdot e}{G_m}\cdot\left(\lambda-t\right)+1\right]\right\}$$

where t (d) is the time of the AD process, G(t) (mL CH_4_/g VS) is the cumulative specific methane production achieved at t (d), G_m_ (mL CH_4_/g VS) and R_m_ (mL CH_4_/g VS/d) are the maximum specific methane production potential and rate estimated by the model, respectively, e = exp (1), and λ (d) is the lag phase time.

The model fitting was performed using the Origin2018 software (OriginLab Corporation, Northampton, USA). The correlation coefficient (r^2^) between experimental and model data was evaluated with the Excel 2016 software (Microsoft Corporation, Redmond, USA).

### Energy assessment

In this study, an energy balance of the whole process was performed using the following hypotheses:

- 1 m^3^ (1 m long by 1 m wide by 1 m high) stainless steel tank (see Fig. S1 of the supplementary material) was used to perform the NMMO pretreatment.
- The tank can treat 90 kg of LRs immersed in 900 kg of NMMO solution, following the substrate-to-solvent ratio used in the present study, i.e. 1:10 (*w/w*).
- The sides and the bottom surface of the tank are thermally insulated with cork layers (thickness = 20 cm). The heat loss through these surfaces is negligible due to the low thermal conductivity of cork, i.e. 0.045 W/(m∙°C) [43].
- The upper surface of the tank is covered with a polyethylene plate (thickness = 3 cm) during the NMMO pretreatment to limit heat loss.
- The tank is already at working temperature (i.e. 120 °C). The ambient temperature is 20 °C.

Under these conditions, the energy required to keep the stainless steel tank at the operating temperature depends on the heat loss from the upper surface to the environment (H_1_) and on the energy used to heat the NMMO solution and the LRs immersed in the tank (H_2_). The two aliquots were calculated using Eq. (2) and Eq. (3):

2 $$H_1=U\cdot A\cdot\frac{\Delta T}{\Delta x}\cdot t_p$$

3 $$H_2=\left(m_{NMMO}\cdot C_{p,NMMO}+m_{LRs}\cdot C_{p,LRs}\right)\cdot\frac{\Delta T}{3600}$$

where U (0.45 W/(m∙°C) [44]) is the thermal conductivity of the upper surface of the tank, A (1 m^2^) is the upper surface of the cubic tank, ∆T (100 °C) is the difference between operating and ambient temperature, ∆x (0.03 m) is the thickness of the insulating plate used to cover the tank, t_p_ (h) is the pretreatment time, m_NMMO_ (900 kg) and m_LRs_ (90 kg) are the masses of the 73% NMMO solution and LRs, respectively, C_p, NMMO_ (3.10 kJ/kg∙°C) is the specific heat capacity of the 73% NMMO solution, calculated considering the C_p_ of water and an 85% NMMO solution [27], C_p, LRs_ (1.20 kJ/kg∙°C [45]) is the specific heat capacity of LRs, and 3600 is the conversion factor between kJ and kWh.

The energy gain (E_p_) from the increment of methane production after the NMMO pretreatment was calculated according to Mancini et al. [6], considering the difference in methane production between pretreated and raw substrates according to Eq. (4). The specific methane potential was rectified using an upscale factor of 0.85 to account for the difference between laboratory and real scale AD conditions [46].

4 $$E_P=\left({SMP}_{pretreated}-{SMP}_{raw}\right)\cdot\xi\cdot CHP$$

where SMP_pretreated_ and SMP_raw_ (kg CH_4_/kg VS) are the specific methane potential from pretreated and raw substrates, ξ is the lower heating value of methane (13.9 kWh/kg CH_4_), and CHP (0.5) is the efficiency of a combined heat and power unit, equal to 50%.

About 85% of the energy used to reach and maintain the pretreatment temperature (i.e. H_1_ + H_2_) can be recovered by heat exchangers [6], accounting for a positive aliquot (E_r,H_) in the energy balance here proposed. The overall energy balance (∆E) is therefore described by Eq. (5):

5 $$\Delta E= {E}_{p}-{H}_{1}-{H}_{2}+{E}_{r, H}$$

### Statistical analysis

The BMP, WRC, and LOI of raw and pretreated substrates were compared by one-way analysis of variance (ANOVA) followed by the Tukey post hoc test. The Pearson correlation coefficient (r) between BMP and pretreatment time, the changes in lignocellulosic composition (i.e. extractives, sugars, and lignin content) and WRC of each substrate were evaluated with the Pearson test. The correlation was considered strong when r was higher than 0.8 [47]. All analyses were performed with Minitab 17 Statistical Software (Minitab LCC, USA). The difference was considered statistically significant when p was < 0.05.

## Results

### Changes in lignocellulosic composition after the NMMO pretreatment

Raw and pretreated substrates were characterised in terms of cellulose and hemicellulose sugars, lignin, and extractive content. The chemical composition analysis (Table 2) showed that raw AS, SCG, and HS have a lignin content of 29.2, 18.8, and 44.2% (based on the dry matter), respectively. Raw AS showed a 42.9% total sugar percentage, mainly constituted by glucan (22.0%) and xylan (18.7%). On the other hand, raw SCG total sugars (42.8%) were primarily composed of mannan (23.5%) and galactan (8.8%), while glucan represented only 8.7% of the overall sugar content. The total sugar percentage of raw HS was lower than that of raw AS and raw SCG, representing only 13.6% of the dry matter, with glucan being the main constituent (10.1%). The compositional analysis also revealed abundant extractives in the raw substrates, in particular for SCG (29.8%) and HS (27.5%).

**Table 2:** Chemical composition of raw and NMMO pretreated substrates expressed as full extractives, structural sugars (glucan, xylan, mannan, arabinan, galactan, and rhamnan), total lignin (Klason lignin and acid soluble lignin), and ashes content. AS: almond shell, SCG: spent coffee grounds, and HS: hazelnut skin. Pretreatment time exposure: 1, 3, and 5 h

Substrate	Full Extractives^a^ (%)	Total Sugars^a, c^ (%)	Total Lignin^a, b^ (%)	Ashes^a^ (%)	Unknown^d^ (%)	Glucan^a^ (%)	Xylan^a^ (%)	Mannan^a^ (%)	Arabinan^a^ (%)	Galactan^a^ (%)	Rhamnan^a^ (%)	Klason Lignin^a^ (%)	Acid Soluble Lignin^a^ (%)
AS raw	10.5 ± 0.1	42.9 ± 0.1	29.2 ± 0.2	2.2 ± 0.1	15.2	22.0 ± 0.3	18.7 ± 0.5	0.1 ± 0.0	0.8 ± 0.0	1.1 ± 0.0	0.3 ± 0.0	27.7 ± 0.2	1.5 ± 0.0
AS 1 h	7.6 ± 0.0	45.9 ± 0.1	30.4 ± 0.1	0.4 ± 0.0	15.6	23.4 ± 0.1	20.5 ± 0.2	0.1 ± 0.0	0.6 ± 0.0	1.0 ± 0.0	0.3 ± 0.0	28.4 ± 0.1	2.1 ± 0.0
AS 3 h	6.3 ± 0.2	45.8 ± 0.7	31.9 ± 0.7	0.7 ± 0.2	15.4	23.7 ± 0.3	20.2 ± 0.4	0.1 ± 0.0	0.6 ± 0.0	1.0 ± 0.0	0.3 ± 0.0	29.8 ± 0.7	2.0 ± 0.0
AS 5 h	5.8 ± 0.2	45.4 ± 0.3	31.3 ± 0.1	0.7 ± 0.0	16.8	24.3 ± 0.2	19.3 ± 0.1	0.0 ± 0.0	0.5 ± 0.0	1.0 ± 0.1	0.3 ± 0.0	29.6 ± 0.1	1.7 ± 0.0
SCG raw	29.8 ± 0.4	42.8 ± 0.1	18.8 ± 0.1	1.3 ± 0.1	7.4	8.7 ± 0.0	0.1 ± 0.0	23.5 ± 0.0	1.5 ± 0.0	8.8 ± 0.1	0.1 ± 0.0	16.8 ± 0.1	1.9 ± 0.0
SCG 1 h	18.7 ± 0.1	54.4 ± 0.2	19.5 ± 0.1	0.7 ± 0.0	6.7	12.1 ± 0.1	0.1 ± 0.0	29.1 ± 0.3	2.0 ± 0.0	11.0 ± 0.0	0.1 ± 0.0	16.8 ± 0.1	2.7 ± 0.0
SCG 3 h	19.9 ± 0.0	52.0 ± 0.3	19.8 ± 0.3	0.6 ± 0.0	7.7	12.2 ± 0.3	0.1 ± 0.0	28.3 ± 0.1	1.8 ± 0.0	9.5 ± 0.0	0.0 ± 0.0	17.7 ± 0.3	2.1 ± 0.0
SCG 5 h	20.5 ± 0.1	53.7 ± 0.4	17.6 ± 0.2	0.9 ± 0.2	7.2	13.8 ± 0.1	0.1 ± 0.1	29.2 ± 0.2	1.7 ± 0.0	8.9 ± 0.0	0.1 ± 0.0	16.1 ± 0.2	1.5 ± 0.1
HS raw	27.5 ± 0.4	13.6 ± 0.3	44.2 ± 0.3	2.6 ± 0.1	12.1	10.1 ± 0.2	0.9 ± 0.0	0.3 ± 0.0	0.7 ± 0.0	0.9 ± 0.0	0.7 ± 0.0	42.7 ± 0.3	1.5 ± 0.0
HS 1 h	23.2 ± 0.2	24.8 ± 0.1	39.0 ± 0.5	2.2 ± 0.0	10.8	18.2 ± 0.1	1.8 ± 0.0	0.5 ± 0.1	1.5 ± 0.0	1.5 ± 0.0	1.3 ± 0.1	37.5 ± 0.4	1.5 ± 0.1
HS 3 h	14.1 ± 0.6	26.6 ± 0.3	44.9 ± 0.3	2.1 ± 0.2	12.2	19.8 ± 0.2	2.3 ± 0.1	0.5 ± 0.1	1.4 ± 0.0	1.4 ± 0.0	1.1 ± 0.0	43.5 ± 0.2	1.5 ± 0.0
HS 5 h	24.7 ± 0.5	28.8 ± 0.5	31.2 ± 0.2	2.2 ± 0.1	13.1	22.1 ± 0.3	2.4 ± 0.1	0.4 ± 0.0	1.4 ± 0.1	1.4 ± 0.1	1.1 ± 0.1	29.5 ± 0.2	1.7 ± 0.0

^*a*^* Based on the dry matter (g/100 g TS)*

^*b*^* Total lignin is calculated as the sum of acid soluble lignin and Klason lignin* [39]

^*c*^* Total sugars are obtained as the sum of glucan, xylan, mannan, arabinan, galactan, and rhamnan*

^*d*^* The unknown matter (e.g. uronic acids, acetyls, and starches) is calculated as the complement to 100 of the other components*

The NMMO treatment affected the composition of the substrates differently (Table 2). AS lost up to 45% of the extractives during the NMMO pretreatment, but no significant effect was observed on the cellulose, hemicellulose, and lignin content. NMMO pretreated SCG showed a higher sugar percentage (+ 27%) compared to the raw SCG, mainly related to glucan (+ 57%). As regards to HS, the NMMO pretreatment removed up to 30% of the total lignin content. Also, the glucan percentage increased by 120% in the most performing pretreatment condition. SCG and HS, respectively, lost up to 37 and 49% of the extractives during the pretreatment.

### Effect of the NMMO pretreatment on external surface area, porosity and crystallinity

**Fig. 1:**
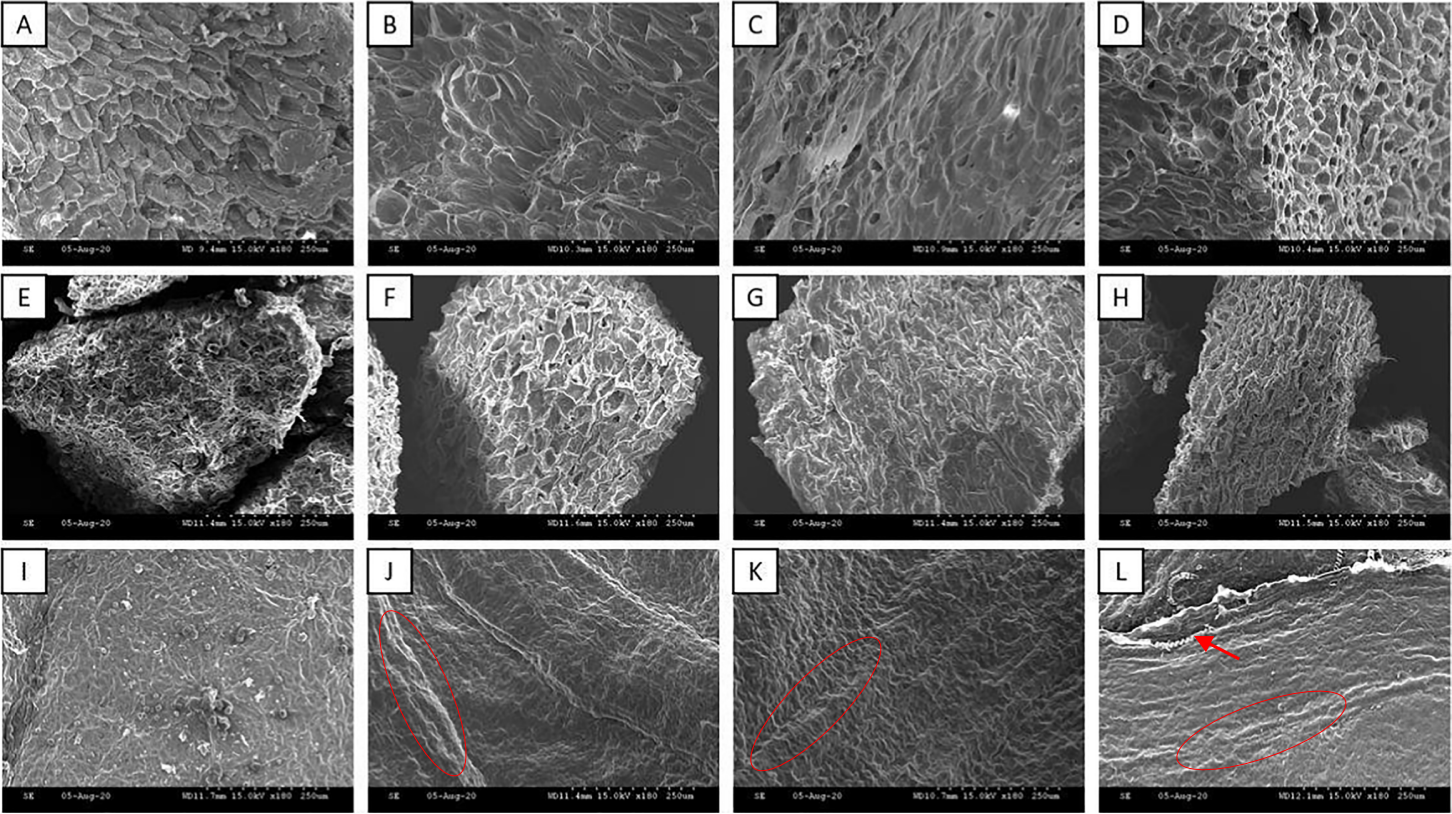
Scanning electron microscopic images of the external surface area of raw and NMMO-pretreated substrates. Raw AS (**A**), 1 h pretreated AS (**B**), 3 h pretreated AS (**C**), and 5 h pretreated AS (**D**). Raw SCG (**E**), 1 h pretreated SCG (**F**), 3 h pretreated SCG (**G**), and 5 h pretreated SCG (**H**). Raw HS (**I**), 1 h pretreated HS (**J**), 3 h pretreated HS (**K**), and 5 h pretreated HS (**L**). AS: almond shell, SCG: spent coffee grounds, and HS: hazelnut skin. Pretreatment time exposure: 1, 3, and 5 h

The SEM images reported in Fig. 1 illustrate the structural changes observed in the external surface between raw and pretreated LRs. Raw AS (Fig. 1A) shows a slivered, hard and compact surface. The NMMO pretreatment appears to be able to smooth the outer surface of AS (Fig. 1B, 1C), removing the upper fraction of the LR, although looking still dense and tough. Figure 1D shows a more porous surface, indicating that the 5 h NMMO pretreatment further changed the external surface of AS. Raw SCG (Fig. 1E) shows a stringy external surface. The NMMO pretreatment thus altered the substrate, which appears cracked with exposed boundaries fractures after the pretreatment (Fig. 1F, 1G, and 1H). On the other hand, among the three substrates HS shows the most appreciable effects in terms of cellulose swelling caused by the NMMO pretreatment. The external surface of raw HS (Fig. 1I) is compact, and the cellulose filaments appear thin and embodied in the lignocellulosic structure. The NMMO pretreatment (Fig. 1J, 1K, and 1L) swelled the filaments, increasing the exposure of the cellulosic part of HS to the enzymatic attack. In particular, the 5 h pretreatment seems able to break down part of the cell wall of HS. Some of the cellulose filaments are more exposed and appear crimped and vulnerable (Fig. 1L).

To further inspect the bioaccessible surface area of the LRs, the WRC of the raw and pretreated substrates was measured, as an indicator of porosity. Table 3 shows that the 5 h NMMO pretreatment significantly increased (*p* < 0.05) the WRC of AS from 0.53 to 0.59 g H_2_O/g TS. On the other hand, all the other pretreatment conditions lowered the AS porosity. The WRC of SCG was significantly higher (*p* < 0.05) after the NMMO pretreatment. The highest porosity was observed for the 3 h NMMO pretreated SCG, increasing the WRC by 63%. Finally, in the case of HS, the porosity significantly increased (*p* < 0.05) proportionally to the pretreatment time. The WRC of HS rose from 1.76 to 2.20 g H_2_O/g TS in the most performing pretreatment condition (i.e. 5 h).

**Table 3:** Water retention capacity (WRC) and lateral order index (LOI) followed by statistical comparison of raw and pretreated substrates with 73% NMMO solution. AS: almond shell, SCG: spent coffee grounds, and HS: hazelnut skin. Pretreatment time exposure: 1, 3, and 5 h

Substrate	WRC (g H_2_O/g TS)	Statistical information ^a^	LOI (A1420/A898)	Statistical information ^a^
AS raw	0.53 ± 0.00	b	2.18 ± 0.24	a
AS 1 h	0.45 ± 0.02	c	1.02 ± 0.03	c
AS 3 h	0.47 ± 0.03	c	1.03 ± 0.02	c
AS 5 h	0.59 ± 0.02	a	1.38 ± 0.04	b
SCG raw	1.12 ± 0.03	c	1.39 ± 0.13	a
SCG 1 h	1.81 ± 0.01	ab	1.57 ± 0.30	a
SCG 3 h	1.83 ± 0.06	a	1.56 ± 0.23	a
SCG 5 h	1.72 ± 0.02	b	1.46 ± 0.06	a
HS raw	1.76 ± 0.04	c	3.89 ± 0.40	a
HS 1 h	1.77 ± 0.05	c	1.38 ± 0.12	b
HS 3 h	2.01 ± 0.06	b	1.31 ± 0.24	b
HS 5 h	2.20 ± 0.11	a	1.40 ± 0.07	b

^a^ Not sharing letters means that the condition was significantly different (*p* < 0.05) with the compared condition

The FTIR spectra were significantly different after the pretreatment (see Fig. S2 of the supplementary material). In particular, the absorbance at 1420 cm^−1^ correlated with the crystalline regions of cellulose, decreased after 1 and 3 h pretreatment of AS, while it raised in the 5 h pretreated AS. On the contrary, all pretreatment conditions increased the absorbance at 898 cm^−1^, indicating an increment in the amorphous cellulose for AS (Fig. S2A). The NMMO pretreatment reduced the crystalline and increased the amorphous regions of HS under all pretreatment conditions tested in this study (Fig. S2C). The analysis of the FTIR spectra shows a significant reduction (*p* < 0.05) of the LOI for AS and HS for all pretreatment conditions tested (Table 3). In contrast, the NMMO pretreatment did not alter the peaks at 1420 and 898 cm^−1^ for SCG (Fig. S2B), and no significant change (*p* > 0.05) in LOI was observed (Table 3).

### Impact of the NMMO pretreatment on methane potential and kinetics

**Fig. 2:**
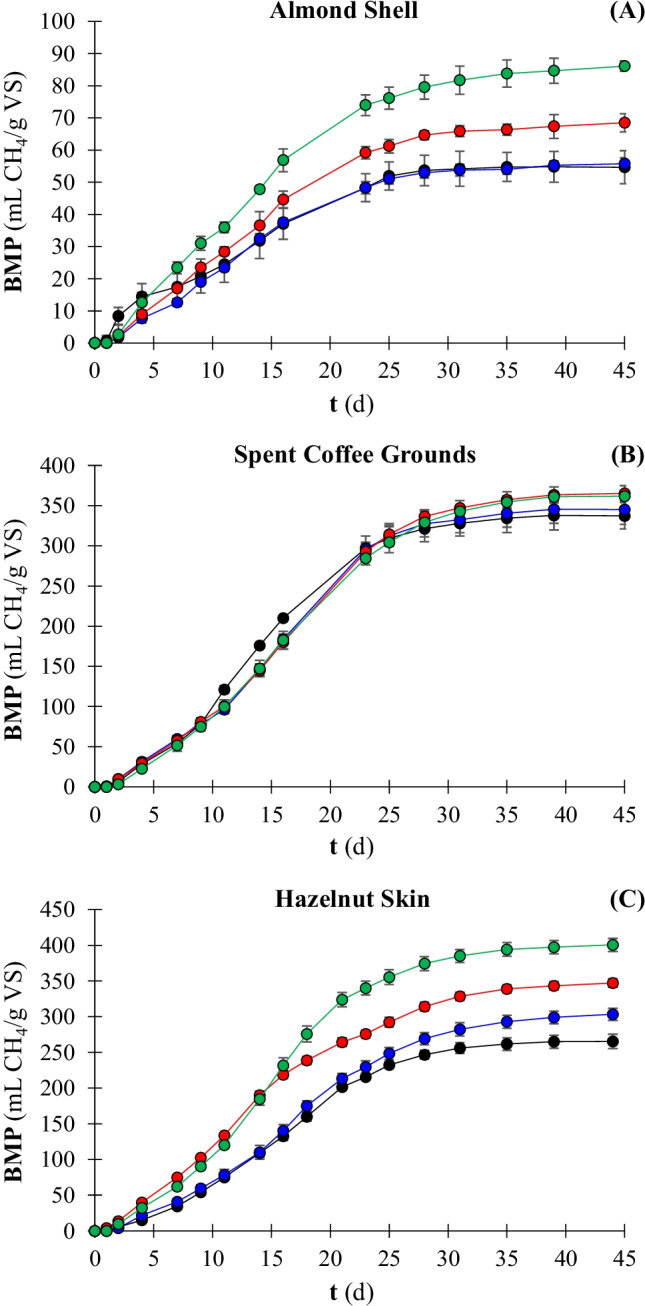
Cumulative methane production from anaerobic digestion of AS (**A**), SCG (**B**), and HS (**C**): untreated (  ), 1 h NMMO (  ), 3 h NMMO (  ), 5 h NMMO (  ) exposure. AS: almond shell, SCG: spent coffee grounds, and HS: hazelnut skin. Pretreatment time exposure: 1, 3, and 5 h

The net cumulative methane production achieved from the AD of raw and NMMO pretreated LRs is given in Table 4. Figure 2 shows the methane production evolution over the 45 days of AD. The AD of untreated LRs showed the high methane potential of raw SCG and raw HS, which reached 337.4 (± 16.5) and 265.4 (± 10.4) mL CH_4_/g VS, respectively. On the other hand, the BMP of raw AS was only 54.7 (± 5.3) mL CH_4_/g VS.

**Table 4:** Biochemical methane potential (BMP) followed by statistical comparison and kinetic parameters, i.e. maximum methane potential (G_m_), maximum methane rate (R_m_), lag phase (λ), and correlation coefficient (r^2^), obtained from the anaerobic digestion process of raw and pretreated substrates with 73% NMMO solution. AS: almond shell, SCG: spent coffee grounds, and HS: hazelnut skin. Pretreatment time exposure: 1, 3, and 5 h

Substrate	BMP (mL CH_4_/g VS)	Statistical information ^a^	Methane production increment (%)	G_m_^b^ (mL CH_4_/g VS)	R_m_^b^ (mL CH_4_/g VS/d)	λ^b^ (d)	r^2c^
AS raw	54.7 ± 5.3	c	-	55.90	2.95	1.7	0.9852
AS 1 h	55.8 ± 2.2	c	2.1	55.81	3.15	3.3	0.9982
AS 3 h	68.5 ± 3.1	b	25.2	68.51	3.76	3.3	0.9970
AS 5 h	86.1 ± 2.0	a	57.5	86.01	4.58	3.0	0.9962
SCG raw	337.4 ± 16.5	a	-	339.69	21.10	5.0	0.9981
SCG 1 h	345.3 ± 18.5	a	2.3	348.22	20.47	5.2	0.9904
SCG 3 h	365.2 ± 9.7	a	8.3	369.52	19.76	5.3	0.9939
SCG 5 h	361.9 ± 4.9	a	7.3	366.84	19.64	5.8	0.9973
HS raw	265.4 ± 10.4	d	-	269.58	14.56	5.4	0.9953
HS 1 h	303.2 ± 9.0	c	14.2	308.97	15.01	5.7	0.9971
HS 3 h	347.1 ± 6.7	b	30.8	351.62	17.30	3.7	0.9972
HS 5 h	400.4 ± 9.5	a	50.9	403.18	23.18	5.1	0.9973

^a^ Not sharing letters means that the condition was significantly different (*p* < 0.05) with the compared condition

^b^ Predicted by fitting the experimental data with a modified Gompertz model

^c^ Correlation coefficient between experimental and model data

The NMMO pretreatment was significantly (*p* < 0.05) effective on AS by increasing the methane potential up to 86.1 (± 2.0) mL CH_4_/g VS (Fig. 2A). The best pretreatment condition corresponded to the longer pretreatment time. Nevertheless, a pretreatment of 3 h also showed an appreciable (*p* < 0.05) enhancement (25%) of the methane production from AS. Similarly, the 3 h and 5 h NMMO pretreatment improved the BMP of HS by 31 and 51%, respectively, reaching a maximum methane potential of 400.4 (± 9.5) mL CH_4_/g VS (Fig. 2C). Regarding HS, the 1 h pretreatment also showed a significant (*p* < 0.05) enhancement in methane production. On the contrary, none of the pretreatment conditions tested in this study was significantly effective on SCG in terms of methane production (Fig. 2B). A slight 8% increase of the BMP was observed for the 3 h and 5 h pretreated SCG, not being statistically significant (*p* > 0.05).

The kinetic analysis showed a high correlation with the modified Gompertz model used to fit the experimental data (Table 4). The model fitting confirmed the pretreatment effectiveness on AS and HS, with the experimental BMP achieving 98% of the maximum methane potential (G_m_) estimated by the model. The 73% NMMO pretreatment enhanced the maximum specific methane production rate (R_m_) of AS from 2.95 to 3.15, 3.76, and 4.58 mL CH_4_/g VS/d for the 1, 3, and 5 h pretreated AS, respectively. However, all the pretreatment conditions increased the lag phase (λ) of AD for AS. The R_m_ of HS increased up to 34% when the NMMO pretreatment lasted 5 h. No significant change of λ was observed by pretreating the HS, apart from the 3 h pretreatment, which resulted in a decreased λ to 3.7 days. Interestingly, the experimental data showed that the NMMO pretreatment led to a delay of the peak of methane production rate only in the case of HS.

### Volatile solid degradation and volatile fatty acids evolution during anaerobic digestion

**Fig. 3:**
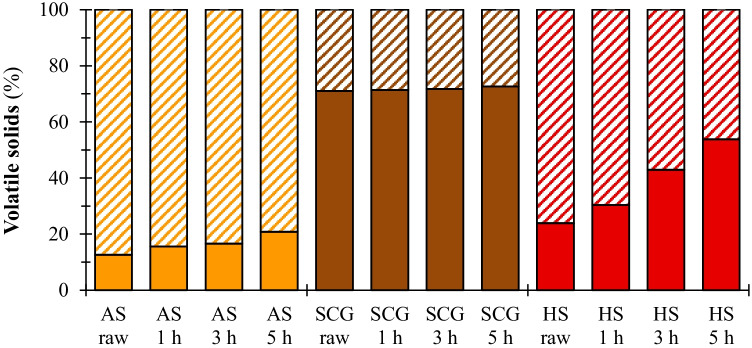
Biodegraded (full bars) and leftover (dashed bars) volatile solids of raw and pretreated substrates after 45 days of anaerobic digestion: AS (  ), SCG (  ), and HS (  ). AS: almond shell, SCG: spent coffee grounds, and HS: hazelnut skin. Pretreatment time exposure: 1, 3, and 5 h

The percentage of VS degraded during the AD process (Fig. 3) accounted for 13, 71, and 24% for raw AS, SCG, and HS, respectively. The pretreatment with 73% NMMO significantly (*p* < 0.05) enhanced the VS biodegradation of AS up to 21%. On the other hand, no significant difference (*p* > 0.05) was observed in VS degraded from raw and pretreated SCG. Regarding HS, all pretreatment durations considerably increased the amount of biodegradable matter (*p* < 0.05), with the increment being positively correlated with the pretreatment time and reaching 54% in the case of 5 h NMMO pretreatment.

**Fig. 4:**
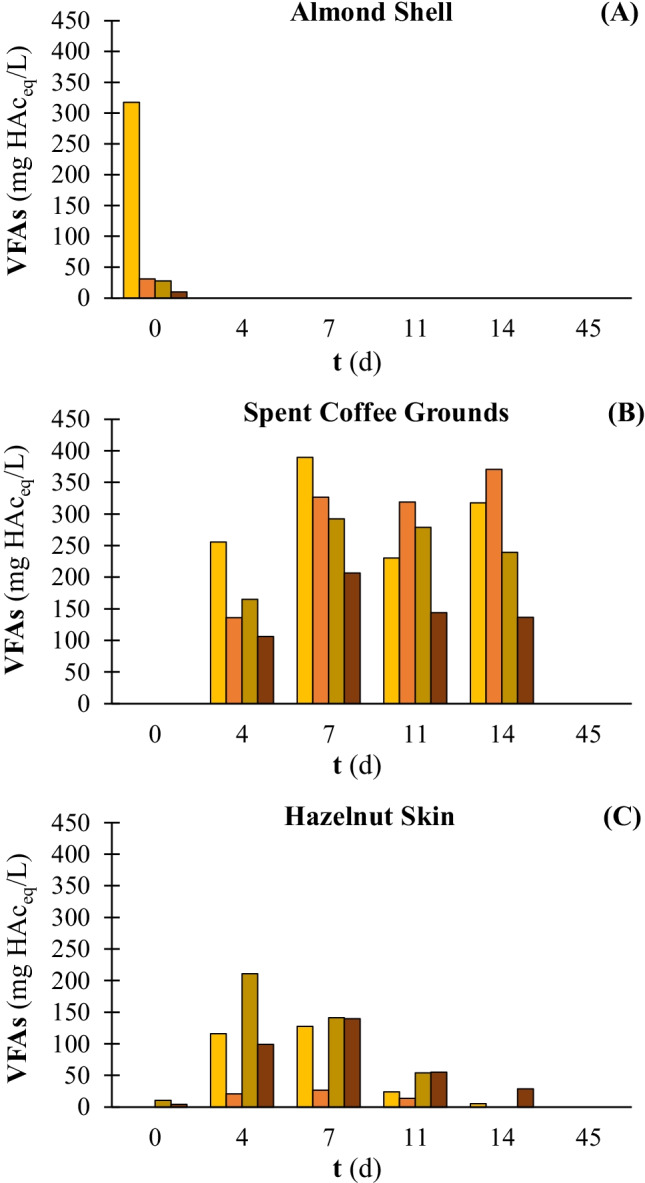
VFAs accumulation during the AD of untreated and pretreated AS (**A**), SCG (**B**), and HS (**C**): untreated  , 1 h NMMO (  ), 3 h NMMO (  ), 5 h NMMO (  ) exposure. AS: almond shell, SCG: spent coffee grounds, and HS: hazelnut skin. Pretreatment time exposure: 1, 3, and 5 h

The VFAs evolution was monitored along with the AD process of raw and pretreated substrates. The total VFAs concentration is reported in Fig. 4 as acetic acid equivalent. Acetic and propionic acids were the main acids produced during the AD of SCG and HS (data not shown). On the contrary, acetic acid was the sole VFA detected during AD of AS (data not shown), which entailed the maximum VFAs concentration on day 0 of the experiment (Fig. 4A). In particular, the VFAs concentration on day 0 of AD of AS was significantly higher when digesting raw (i.e. 317 mg HAc_eq_/L) rather than pretreated (i.e. 31 mg HAc_eq_/L) substrates. The VFAs evolution was similar for raw and pretreated SCG (Fig. 4B). The maximum concentration was observed on day 7 for untreated (i.e. 390 mg HAc_eq_/L), 3 h (i.e. 292 mg HAc_eq_/L) and 5 h (i.e. 207 mg HAc_eq_/L) NMMO pretreated SCG. On the other hand, in the case of 1 h NMMO pretreated SCG, the VFAs concentration was almost stable between day 7 (i.e. 327 mg HAc_eq_/L) and 14 (i.e. 371 mg HAc_eq_/L). As regards HS (Fig. 4C), the highest VFAs accumulation was observed on day 4 for the 3 h NMMO pretreated HS (i.e. 211 mg HAc_eq_/L), while the peak was obtained on day 7 for the other pretreatment conditions. The VFAs concentration approached zero already on day 14 and was null at the end of the AD process, i.e. on day 45.

### Energy saving

The energy balance performed in this study (Table 5) revealed the feasibility of applying the NMMO pretreatment for HS, giving an energy gain of 0.18 and 0.40 kWh/kg VS after 3 h and 5 h pretreatment, respectively. On the other hand, the energy assessment returned a negative energy balance for pretreated AS and SCG.

**Table 5:** Energy balance (∆E) calculated considering energy costs (H_1_ and H_2_), energy recovered by heat exchangers (E_r, H_), and energy gain from the extra methane produced (E_P_) from pretreated substrates. AS: almond shell, SCG: spent coffee grounds, and HS: hazelnut skin. Pretreatment time exposure (t_p_): 1, 3, and 5 h

Substrate	t_p_ (h)	H_1_ (kWh)	H_2_ (kWh)	E_r, H_ (kWh)	E_P_ (kWh)	∆E (kWh)	∆E (kWh/kg VS)
AS 1 h	1	1.5	80.47	69.67	0.38	-11.92	-0.15
AS 3 h	3	4.5	80.47	72.22	4.63	-8.12	-0.10
AS 5 h	5	7.5	80.47	74.77	10.55	-2.64	-0.03
SCG 1 h	1	1.5	80.47	69.67	2.57	-9.72	-0.13
SCG 3 h	3	4.5	80.47	72.22	9.09	-3.65	-0.05
SCG 5 h	5	7.5	80.47	74.77	8.00	-5.19	-0.07
HS 1 h	1	1.5	80.47	69.67	12.46	0.17	0.00
HS 3 h	3	4.5	80.47	72.22	26.95	14.20	0.18
HS 5 h	5	7.5	80.47	74.77	44.52	31.32	0.40

## Discussion

### Anaerobic digestion of untreated almond shells, spent coffee grounds, and hazelnut skin

The AD of the three raw LRs under investigation showed that SCG and HS had a high methane potential compared to most studied agricultural and industrial LRs [17, 48], producing 337.4 and 265.4 mL CH_4_/g VS, respectively (Table 4). On the other hand, raw AS only produced 54.7 mL CH_4_/g VS (Table 4), being in the range of methane production from that of other nut shells observed by Shen et al. [17]. The trend in methane production shows that, after a lag phase in which the microorganisms hydrolysed most of the biodegradable matter, the saturation in methane production was reached around day 30 of AD, regardless of the substrate (Fig. 2). This trend indicates that the solubilised biodegradable matter was easily converted to methane, with acetic acid being the main component in the intermediately produced VFAs mixture [49].

One of the most important factors hindering the AD of LRs is the lignin content [9]. The three substrates used in this study showed different chemical compositions, but all have a rather high lignin content (Table 2), i.e. 29.2, 18.8 and, 44.2% (based on the dry matter), respectively, for AS, SCG, and HS. Based on the lignin content only, HS was expected to be the most recalcitrant substrate among the three. Nevertheless, the experimental evidence showed that many other factors, i.e. porosity, external surface, crystallinity, and extractives content, affect the AD of LRs.

AS was indeed the least suitable substrate for AD, resulting in the lowest methane production among the raw substrates (Fig. 2). This result is consistent with previous studies, where a methane production of 45.4 (± 8.7) mL CH_4_/g VS was achieved [17]. Other studies reported an even lower methane potential of AS, i.e. 20.2 (± 13.0) mL CH_4_/g VS [50] and 23.2 (± 9.6) mL CH_4_/g VS [37]. The low methane potential did not reflect the cellulose (22.0%) and hemicellulose (21.0%) content of the AS here used, suggesting a greater potential of AS for AD [51]. Nevertheless, the scarce WRC (0.53 g H_2_O/g TS), the high LOI (i.e. 2.18), and the hard external surface of AS (Table 3 and Fig. 1A) most likely prevented the microorganisms to attack the substrate, resulting in slow and inefficient AD [52]. The VFAs evolution observed in the present study (Fig. 4A) suggests that AS has a remarkable aliquot of extractives easily soluble in aqueous solution, which immediately hydrolysed and were likely converted into VFAs peaking at 317.3 (± 24.0) mg HAc_eq_/L on day 0 (Fig. 4A). On the other hand, the absence of VFAs accumulated during the subsequent days of AD indicates that the hydrolysis of cellulose and hemicellulose from AS is slow, with methanogenic archaea acting at the same speed of hydrolytic and acidogenic bacteria [3].

On the contrary, SCG and HS showed a higher methane potential (Table 4). In particular, in this study, HS produced 265.4 (± 10.4) mL CH_4_/g VS. This result is comparable with previous studies where the same substrate was used [6, 27, 29]. A significantly lower methane production was obtained from HS (i.e. 17.3 mL CH_4_/g VS) when using a granular sludge as the source of microorganisms [37]. This evidence highlighted that not only the physical and chemical characteristics of the substrate but also the type of inoculum greatly affects the AD process, as previously observed by Gu et al. [53] for rice straw. The methane obtained from SCG was 337.4 (± 16.5) mL CH_4_/g VS. This value is comparable with the available literature regarding the AD of SCG under similar operative conditions [54, 55]. Contrary to HS, the AD of SCG seems to be less susceptible to the type of inoculum since no significant difference was observed with a previous study where a granular sludge was used [37]. The VFAs analysis (Fig. 4) reflected the usual trend of LRs, with slow hydrolysis and maximum VFAs accumulation after a few days of AD [56]. In particular, the maximum VFAs accumulation was observed on day 7, with a concentration of 389.9 (± 33.2) and 127.4 (± 86.7) mg HAc_eq_/L for SCG and HS, respectively.

The higher biodegradability of SCG and HS is due to the physical characteristics of the substrates. SCG and HS showed a significantly higher WRC than AS (Table 3). The LOI indicates that mainly crystalline cellulose prevails in HS, while SCG is composed of both crystalline and amorphous cellulose (Table 3). Besides, the external surface of SCG and HS appeared smoother than that of AS (Fig. 1). In addition, the content of extractives (Table 2) may also have positively influenced the AD process since these compounds also include easily biodegradable matter, e.g. free sugars. The results obtained in this study are in accordance with previous works [57], where the porosity and other physical characteristics of LRs were key factors for efficient AD [58]. As a further aspect, the measurement of the leftover VS at the end of the experiment confirmed the recalcitrance of AS and HS (Fig. 3). Only 12.6 and 32.7% of the overall volatile matter was degraded after 45 days of AD for AS and HS, respectively (Fig. 3). On the other hand, 71.1% of the VS from SCG was degraded during the AD process (Fig. 3).

### NMMO pretreatment effectiveness on lignocellulosic substrates

#### Almond shell

The pretreatment with a 73% NMMO solution was effective on AS, achieving the maximum methane potential (86.1 mL CH_4_/g VS) from the 5 h pretreated AS and increased by 58% compared with the raw AS (Table 3). The effect of the pretreatment increased with its duration (Fig. 2A), showing a strong direct correlation, i.e. r = 0.980 (see Table S1 of the supplementary material). Nevertheless, the maximum methane production obtained in this study is still far from the theoretical methane potential of AS, i.e. 490 mL CH_4_/g VS [37]. The low methane production is reflected by the limited VS degradation (Fig. 3). The highest VS degradation (20.8%) occurred for the 5 h NMMO pretreated AS after 45 days of AD, meaning that the microorganisms did not degrade most of the available VS. The non-degraded VS (Fig. 3) certainly includes lignin, which represents 31.3% of the 5 h pretreated AS composition (Table 2). Only fungi and specific strains of bacteria are able to decompose lignin thanks to their selective enzymatic system [59, 60]. Thus, it is very likely that most of the initial lignin content remained unaltered after AD [61], eventually accounting for non-degraded VS, which is one of the aspects contributing to the low methane potential of raw and pretreated AS.

The NMMO pretreatment did not lead to significant changes in sugars and lignin content of AS (Table 2), in line with the results obtained for other substrates such as flower waste [62] and wheat straw [52]. Thus, the enhanced methane production observed with the pretreated substrate is attributed to other aspects. In particular, the NMMO pretreatment reduced the ratio between amorphous and crystalline cellulose, i.e. LOI, under all pretreatment conditions, indicating a higher biodegradability of the pretreated AS [63]. Also, the WRC of AS increased from 0.53 to 0.59 g H_2_O/g TS after 5 h of pretreatment (Table 3). Mancini et al. [52] obtained similar results for wheat straw using an 85% NMMO pretreatment for 3 h. In that study, WRC increased from 1.30 to 1.90 g H_2_O/g TS resulting in an 11% increment in methane production. On the other hand, in the present study, no effect was observed on the 3 h and 1 h pretreated AS. The increment in methane production from 3 h pretreated AS (i.e. 25.2%) might be, therefore, associated with the strength of the cellulose-hemicellulose-lignin linkage that is likely weakened by the NMMO pretreatment, as previously observed by Cheng et al. [26] for cassava residues. In addition, a moderate inverse correlation, i.e. r = –0.776, was observed between the extractives content and methane potential of AS (see Table S1 of the supplementary material). This correlation could be due to inhibitory compounds initially present in the extractives of AS that were lost during the pretreatment [64].

Although the outer surface of the pretreated AS is smoother than for the raw substrate (Fig. 1A-D), it looks resistant and leathery, confirming that the pretreatment did not significantly alter the physical structure of the substrate. In a previous study, Oliva et al. [37] investigated the effectiveness of methanol-organosolv pretreatment on AS. In that case, the pretreatment affected neither the external surface nor the porosity of the substrate, resulting in no increment in the methane production. A longer NMMO pretreatment or a different, more aggressive pretreatment, such as acid or alkaline pretreatment, may be tested to disrupt the hard and compact structure of AS. Nevertheless, previous studies reported that longer NMMO pretreatment may result in loss of hemicellulose sugars [65, 66]. Overall, the low porosity and the highly resistant outer surface, together with the high lignin content, explain the low methane potential of raw and pretreated AS.

As regards the trend of VFAs (Fig. 4A), the higher concentration observed on day 0 reflects the methane production of the following days (1 – 4) from raw AS. Methane production from raw AS was higher compared to the pretreated AS (Fig. 2A) until day 4, resulting in a shorter lag phase (Table 4). The lower VFAs concentration observed on day 0 in the bottles with the pretreated AS was likely due to the loss of non-structural sugars during NMMO pretreatment. Acidogenic bacteria can easily convert free sugars in VFAs, allowing faster methane production in the first days of AD [67]. The failure to accumulate VFAs during the AD progress probably suggests that the hydrolysis rate was still low, despite the NMMO pretreatment enhancing the VS biodegradability of AS (Fig. 3).

#### Spent coffee grounds

The cumulative methane production obtained from SCG was similar for the raw and pretreated substrates (Fig. 2B), showing that NMMO pretreatment was ineffective for SCG. In this study, depending on the pretreatment condition, the methane potential of SCG ranged between 337.4 and 365.3 mL CH_4_/g VS (Table 4). Several studies focused on SCG for biofuels or valuable biomolecules production [68, 69]. Nevertheless, only Girotto et al. [54] reported a methane production slightly higher than that here obtained, showing that an 8% NaOH pretreatment allowed to produce 392 mL CH_4_/g VS from SCG. The VFAs evolution (Fig. 4B) follows the typical trend with a peak within the first 10 days of the process followed by a gradual decrease in their concentration [70]. The maximum VFAs concentration was observed on day 7 (Fig. 4) and is significantly below the overall VFAs inhibitory threshold of 6000 mg/L [71].

The WRC of SCG significantly increased after the NMMO pretreatment (Table 3), with the maximum porosity (i.e. 1.83 g H_2_O/g TS) corresponding to the 3 h NMMO-pretreated SCG. This is in agreement with the increment of porosity reported by Shafiei et al. [65] for pinewood. In addition, the pretreatment allowed increasing the sugar percentage by up to 25% (mainly glucan and mannan) along with the pretreatment duration (Table 2) due to the loss of other components, i.e. extractives. Teghammar et al. [66] obtained similar results performing an 85% NMMO pretreatment on spruce and triticale straw, increasing the methane potential of these substrates but also observing a loss of hemicellulose sugars when increasing the pretreatment time. On the other hand, Teghammar et al. [66] showed that pretreatment times longer than 1 h reduced the glucan content and lowered the methane potential of rice straw. On the contrary, in the present study, the higher sugar percentage achieved with NMMO pretreatment did not affect the methane production from SCG. The loss of extractives from 30 to 37% during the pretreatment can explain this result. SCG are rich in free sugars, proteins and fatty acids that microorganisms can easily convert into methane under anaerobic conditions. The loss of these molecules most probably reduced the methane potential of SCG [69].

The ineffectiveness of the NMMO pretreatment on SCG is also linked to the high VS degradation observed for raw SCG. In fact, despite the considerable lignin percentage (i.e. 18.8%), 71.1% of the initial VS embedded in the raw SCG was degraded after AD, and the rate of VS degradation did not significantly increase after NMMO pretreatment (Fig. 3). The non-degraded solids include lignin, which barely changed after the NMMO pretreatment (Table 2). The VS degradation rate observed for SCG is comparable with the result reported by Li et al. [72] for a much easier biodegradable substrate, i.e. food waste. The lignin content and the VS degradation might suggest that not much further methane potential can be gained from the investigated SCG. An alternative approach can lead to a better utilisation of the single component of this substrate, by, for instance, extracting valuable components from SCG before subjecting it to any pretreatment. The cascade approach would allow recovering molecules with high commercial value while providing a simpler substrate for AD [73].

#### Hazelnut skin

The BMP of HS increased from 265.5 up to 400.4 mL CH_4_/g VS after the NMMO pretreatment. The effectiveness is strongly correlated (r = 0.996) with the pretreatment time, with the 5 h NMMO pretreatment enhancing methane production up to 51% (Table 4 and Fig. 2C). The increased total sugar content (r = 0.886) (Table 2) and WRC (r = 0.951) (Table 3) of the pretreated HS were strongly correlated with the increase in methane potential (see Table S1 of the supplementary material), following the results obtained by Kabir et al. [25] with barley straw and a pretreatment time of 7 h. The LOI of HS decreased from 3.90 to approximately 1.40, regardless of the pretreatment exposure, as previously observed by Purwandari et al. [63]. Moreover, the increased VS degradation (Fig. 3) reflected the enhanced BMP achieved after the pretreatment.

The NMMO pretreatment altered the external surface of HS and exposed the swelled cellulose filaments, as illustrated in Fig. 1J, 1K, and 1L. Similarly, the NMMO pretreatment was reported to be able to change the external surface of pinewood and oil palm empty fruit bunch enhancing the bioavailability of the cellulosic component of the LRs [63, 65]. The chemical structure of NMMO presents weak N–O polar bonds that can be easily broken to form new hydrogen bonds with cellulose in aqueous solutions. The NMMO solution penetrates the cell wall, increases its internal osmotic pressure, and expands the cellulosic fibres creating balloons. Inside the balloons, depending on the NMMO concentration and characteristics of the substrates, cellulose dissolution can occur. When the osmotic pressure exceeds the membrane resistance, the balloons explode, thus releasing dissolved cellulose [24].

The VFAs evolution (Fig. 4C) revealed that the highest concentration (i.e. 211 mg HAc_eq_/L) was observed at day 4 and corresponded to the 3-h pretreated HS, which was the pretreatment condition showing the best performance in terms of methane production at that time of the AD process (Fig. 2C). After day 4, the VFAs concentration in the same bottles decreased and reflected the drop of methane production observed after day 14.

Mancini et al. [27] previously studied the effectiveness of NMMO pretreatment on HS under dissolution mode conditions (i.e. a NMMO concentration of 85%). In that case, no significant difference in methane production was observed between raw and pretreated HS. On the contrary, the swelling mode (i.e. 73%) was effective under all pretreatment conditions in the present study. This confirmed the result obtained with cotton by Jeihanipour et al. [20], who observed an increased BMP only using 73 and 79% NMMO solutions during the pretreatment. Furthermore, Purwandari et al. [63] showed that a 1 h 73% NMMO pretreatment was more effective than that performed at 85% for oil palm empty fruit bunch.

The effectiveness of the NMMO pretreatment here performed is attributable to lignin removal and, consequently, an increased sugar percentage in the pretreated substrates (up to 112%). A moderate inverse correlation, i.e. r = –0.708, was observed between the lignin content and methane production (see Table S1 of the supplementary material). Although lignin attack is not an expected effect of NMMO pretreatment [23], a long exposure time (i.e. 5 h) at high temperature (i.e. 120 °C) reduced the lignin percentage in HS by 29%. Other authors previously reported a significant lignin removal upon performing 75% NMMO pretreatment for 15 h, while shorter pretreatments using 85% NMMO solution did not remarkably affect the lignin content of forest residues [74]. Teghammar et al. [66] reported a 34% lignin removal from triticale straw after a 15 h pretreatment with 85% NMMO solution, but no effect on the lignin content was observed when using a shorter pretreatment time. On the other hand, other authors did not report any change in lignin content from forest residues and barley straw after 30 h pretreatment using an 85% NMMO solution [25]. The results of the present study and the available literature suggest that lignin removal during NMMO pretreatment mainly depends on the specific characteristics of the substrate and is more likely to occur when performing the pretreatment at lower NMMO concentrations.

To the best of the authors’ knowledge, the present work is the first article showing significant delignification (i.e. up to 29%) of highly lignified materials after NMMO pretreatment. Kabir et al. reported only a 7% lignin removal from forest residues after 15 h pretreatment [75]. The effectiveness of lignin removal can be related to the high WRC of raw HS (i.e. 1.76 g H_2_O/g TS), which allowed the solvent to penetrate the substrate faster than in other LRs [37]. Unfortunately, none of the authors who observed lignin removal after NMMO pretreatment reported substrate characterisation in terms of porosity. Thus, this hypothesis still requires confirmation with further studies.

The content and type of extractives also influence the biodegradability of LRs [64]. Extractives include primary substrates for the AD process, such as non-structural sugars, proteins and fats, but also phenolic compounds, which negatively affected the AD of LRs [64, 76]. In particular, Kayembe et al. [77] showed that the number of hydroxyl groups on the aromatic compounds is inversely related to the toxicity of the phenolic monomers during AD. HS is indeed an extractive-rich LR (Table 2), with polyphenols representing 7% of the overall composition [78]. A selective polyphenols removal from HS before AD can, thus, provide the dual benefit of recovering valuable compounds and removing inhibitors for the subsequent valorisation process [79–81].

### Scale-up perspective of the NMMO pretreatment: economical, energetic and environmental remarks

In the present study, the NMMO pretreatment under improved operating conditions enhanced the methane potential of AS and HS (Table 4). Nevertheless, a preliminary energy assessment demonstrated that a considerable extra methane production is required to counterbalance the pretreatment costs. This analysis showed that only the NMMO pretreated HS led to an energetic advantage (i.e. ∆E = 0.40 kWh/kg VS, at best) in the bioconversion process (Table 5). On the other hand, Mancini et al. [27] did not achieve any energy gain by treating the same substrate with an 85% NMMO solution.

The energy gain obtained in the present study can be theoretically extended to the global production of hazelnuts (i.e. 512,100 tons/year [15]), considering a correction factor of 0.03 to take into account the percentage (*w/w*) of HS in the whole fruit [78]. A preliminary economic evaluation considering the energy average world price of 0.14 $/kWh [82] estimates an economic gain of roughly 75 million $/year by pretreating the HS under the operating conditions proposed in the present study.

A preliminary energetic and economic analysis is essential to evaluate the feasibility of using the NMMO pretreatment. Nevertheless, when evaluating the implementation of the pretreatment on an industrial scale, further aspects should be considered. For instance, the washing of the LRs and the recovery and reuse of the solvent are crucial aspects to reduce the costs of NMMO pretreatment. The cost of the NMMO, i.e. 4 €/kg [83], is one of the factors limiting the NMMO pretreatment application on an industrial scale. Nevertheless, up to 99% of the NMMO can be recovered by evaporating the extra water used to wash the LRs [19]. Shafiei et al. [84] showed that multistage evaporation units are up to 80% more efficient than a single stage for energy savings. In particular, the costs for water evaporation greatly increase when concentrating NMMO from 70 to 86% [84]. The strong hydrogen bonds between water and NMMO require a further elevation of the evaporating temperature by 30 °C to obtain the 86% NMMO solution [84], increasing the process costs and the risk for NMMO degradation and side reactions [85]. Therefore, using the 73% NMMO solution proposed in the present study rather than the most commonly investigated 85% NMMO solution for LRs pretreatment could offset the overall costs of the NMMO pretreatment process.

The effectiveness of recovered NMMO is still debated and seems to be related to the initial chemical composition of the LR. Recovered NMMO was effective on pure cellulose and barley straw [20, 25]. On the other hand, the effectiveness was up to 55% lower for forest residues [25]. The lower performance of recovered NMMO seems to be related to the presence of extractives such as tannins, phenols and acid resins hydrolysed during the pretreatment. Therefore, the suggestion of recovering these compounds before pretreating the substrates for AD is furtherly endorsed. It is also fair to point out that Kabir et al. [25] performed a much longer (i.e. 30 h) NMMO pretreatment compared to that of the present study, and the use of propyl gallate to stabilise the reaction was not reported in that study. Therefore, the failure of reusing the NMMO solution shown by Kabir et al. [25] for forest residues is likely to be due to the solvent degradation caused by side reactions occurring during the pretreatment [85].

The techno-economic study proposed by Teghammar et al. [83] revealed that the amount of LRs treated by the NMMO unit is another crucial aspect of the process. In that study, the pretreatment of at least 50000 tons (dried weight) of forest residues per year allowed an efficient NMMO pretreatment. Apart from the economic perspective, the environmental impact is a critical aspect when dealing with the pretreatment of LRs. In particular, NMMO pretreatment was compared with steam explosion via life cycle assessment, showing that the bioenergy gain due to NMMO pretreatment is more environmentally sustainable in terms of resources, climate change, ecosystem quality, and human health [86].

To the best of the authors’ knowledge, the NMMO pretreatment has not yet been implemented on an industrial scale for LRs pretreatment. Nevertheless, in that perspective, using a less concentrated NMMO solution would make the process for LRs pretreatment more similar to the Lyocell process, where the NMMO concentrations range from 60 to 75% [24], which means working with technologies already employed on an industrial scale.

## Conclusion

Swelling mode (i.e. 73%) NMMO pretreatment is an effective technique to increase the methane potential of AS and HS. The pretreatment time was a key parameter, resulting in different effects on chemical composition, physical characteristics, and methane potential of the LRs involved in the study. Of the three LRs, AS and HS were positively impacted after pretreatment improving the extraction of potential energy through methane production by 58 and 51%, respectively. The NMMO pretreatment increased the BMP of AS up to 86.1 mL CH_4_/g VS. Nevertheless, the energy balance revealed that the extra methane produced did not compensate for the pretreatment costs. No significant change in the BMP of SCG was observed, despite the higher sugar percentage and WRC. On the other hand, NMMO pretreatment enhanced the AD from HS, increasing the methane production by 14, 31, and 51% after 1, 3, and 5 h pretreatment, respectively. The methane gain was the consequence of an increased sugar concentration, lower lignin content and LOI, and higher porosity. In addition, the loss of phenolic compounds may have positively influenced the AD process. The energy balance revealed that the NMMO pretreatment is attractive for HS, showing a positive energy gain of 0.18 and 0.40 kWh/kg VS for 3 h and 5 h pretreated HS, respectively. This study opened new perspectives for the valorisation of emerging LRs, such as nut residues. In particular, the abundance of extractives in the LRs here investigated is thus far an understudied aspect and will benefit from further studies on their role in AD.

## Declaration of interest statement

The authors declare that they have no known competing financial interests or personal relationships that could have appeared to influence the work reported in this paper.

## CRediT author statement

A. Oliva: conceptualisation, data curation, formal analysis, investigation, validation, visualisation, writing—original draft, and writing—review & editing. L. C. Tan: supervision and writing—review & editing. S. Papirio: supervision, resources, writing—review & editing, and project administration. G. Esposito: supervision, resources, and writing—review & editing. P. N. L. Lens: supervision, resources, writing—review & editing, project administration, and funding acquisition.

### Supplementary Information

Below is the link to the electronic supplementary material.

Supplementary file1 (DOCX 25 KB)
