# Supplementary material for: Use of N-Methylmorpholine N-oxide (NMMO) pretreatment to enhance the bioconversion of lignocellulosic residues to methane
Source: Biomass Convers Biorefin. 2022 Aug 17;14(10):11113–30. doi: 10.1007/s13399-022-03173-x (PMC11060973; doi:10.1007/s13399-022-03173-x)
Supplement: Supplementary file 1 — Supplementary file1 (DOCX 25 KB) [file 13399_2022_3173_MOESM1_ESM.docx]

# Supporting information accompanying the manuscript:

# Ultrasounds application for nut and coffee wastes valorisation via biomolecules solubilisation and methane production

A. Oliva^1*^, S. Papirio^2^, G. Esposito^2^, P. N. L. Lens^1^

^1^National University of Ireland Galway, University Road, H91 TK33, Galway, Ireland

^2^University of Naples Federico II, Department of Civil, Architectural and Environmental Engineering, Via Claudio 21, 80125, Naples, Italy

*Corresponding author

Email: [A.OLIVA1@nuigalway.ie](mailto:A.OLIVA1@nuigalway.ie)

**Table S1 –** pH measured in the bottles digesting the liquid fraction from ultrasounds pretreatment at day 0 of observation and methane potential with related statistical information of the liquid fractions expressed either as methane per 100 mL of liquor or as methane per gram of glucose added from the liquor.

| **Substrate** | **Pretreatment condition** | **Initial pH** | **Methane production**  (mL/100 mL liquor) | **Statistical information ^a^** | **Liquor_in_**  (mL) | **Glucose_in_** (g) | **Methane potential**  (mL/g glucose_in_) | **Statistical information ^a^** |
| --- | --- | --- | --- | --- | --- | --- | --- | --- |
| Hazelnut Skin  liquor | H_2_O T_amb_ | 8.0 ± 0.0 | 85.3 ± 12.2 | a | 30 | 0.19 | 131.5 ± 18.7 | a |
|  | H_2_O 80 °C | 7.9 ± 0.1 | 79.9 ± 5.6 | a | 30 | 0.30 | 80.8 ± 5.6 | b |
|  | MeOH T_amb_ | 7.8 ± 0.1 | 0.0 ± 2.0 | b | 30 | 0.39 | 0.0 ± 1.6 | c |
|  | MeOH 80 °C | 7.9 ± 0.1 | 0.0 ± 0.7 | b | 30 | 0.40 | 0.0 ± 0.6 | c |
| Almond Shell liquor | H_2_O T_amb_ | 7.9 ± 0.1 | 84.4 ± 2.6 | b | 30 | 0.06 | 431.6 ± 13.2 | a |
|  | H_2_O 80 °C | 7.9 ± 0.1 | 107.0 ± 6.2 | a | 30 | 0.07 | 434.2 ± 25.1 | a |
|  | MeOH T_amb_ | 7.7 ± 0.1 | 0.0 ± 0.5 | c | 30 | 0.06 | 0.0 ± 2.3 | b |
|  | MeOH 80 °C | 7.7 ± 0.1 | 0.0 ± 0.6 | c | 30 | 0.09 | 0.0 ± 2.1 | b |
| Spent Coffee Grounds liquor | H_2_O T_amb_ | 7.9 ± 0.1 | 102.9 ± 5.9 | b | 30 | 0.05 | 685.5 ± 39.5 | a |
|  | H_2_O 80 °C | 7.9 ± 0.0 | 160.9 ± 16.6 | a | 30 | 0.08 | 590.5 ± 60.8 | a |
|  | MeOH T_amb_ | 7.7 ± 0.1 | 0.0 ± 0.5 | c | 30 | 0.05 | 0.0 ± 2.7 | b |
|  | MeOH 80 °C | 7.8 ± 0.1 | 1.2 ± 0.1 | c | 30 | 0.06 | 6.1 ± 0.6 | b |

^a^ Not sharing letters means that the condition was significantly different (p < 0.05) than the compared condition.

**Table S2** – Mass balance assessment considering the full extractives, total structural sugars, total lignin, ashes, and unknown matter measured before and after ultrasounds pretreatment.

| **Substrate** | **Pretreatment Condition** | **Initial substrate**  (g) | **Substrate solubilisation** (%) | **Substrate loss** (g) | **Solid fraction recovered**  (g) | **Full Extractives** (g) | **Total Structural**  **Sugars**  (g) | **Total Lignin**  (g) | **Ashes** (g) | **Unknown** (g) |
| --- | --- | --- | --- | --- | --- | --- | --- | --- | --- | --- |
|  |  |  |  |  |  |  |  |  |  |  |
|  |  |  |  |  |  |  |  |  |  |  |
| Hazelnut Skin | Raw | 15 | 0 | 0.0 | 15.0 | 5.3 | 2.1 | 5.9 | 0.4 | 1.3 |
|  | H_2_O_T_amb_ | 15 | 14.60 | 2.2 | 12.8 | 4.4 | 1.8 | 4.6 | 0.3 | 1.8 |
|  | H_2_O_80 °C | 15 | 19.33 | 2.9 | 12.1 | 3.9 | 2.1 | 4.7 | 0.2 | 1.3 |
|  | MeOH_T_amb_ | 15 | 17.16 | 2.6 | 12.4 | 4.2 | 1.8 | 4.5 | 0.2 | 1.7 |
|  | MeOH_80 °C | 15 | 23.51 | 3.5 | 11.5 | 3.5 | 2.0 | 4.2 | 0.2 | 1.7 |
| Almond Shell | Raw | 15 | 0.00 | 0.0 | 15.0 | 1.1 | 6.2 | 5.6 | 0.2 | 1.9 |
|  | H_2_O_T_amb_ | 15 | 5.74 | 0.9 | 14.1 | 0.5 | 5.7 | 5.9 | 0.1 | 1.9 |
|  | H_2_O_80 °C | 15 | 5.61 | 0.8 | 14.2 | 0.9 | 6.2 | 5.5 | 0.0 | 1.5 |
|  | MeOH_T_amb_ | 15 | 5.61 | 0.8 | 14.2 | 0.5 | 6.3 | 5.8 | 0.1 | 1.4 |
|  | MeOH_80 °C | 15 | 6.42 | 1.0 | 14.0 | 0.6 | 5.7 | 5.9 | 0.0 | 1.9 |
| Spent Coffee Grounds | Raw | 15 | 0.00 | 0.0 | 15.0 | 4.3 | 6.5 | 2.8 | 0.2 | 1.1 |
|  | H_2_O_T_amb_ | 15 | 15.36 | 2.3 | 12.7 | 3.1 | 6.0 | 2.7 | 0.1 | 0.8 |
|  | H_2_O_80 °C | 15 | 15.15 | 2.3 | 12.7 | 3.3 | 5.5 | 2.7 | 0.1 | 1.1 |
|  | MeOH_T_amb_ | 15 | 20.75 | 3.1 | 11.9 | 2.8 | 5.8 | 2.5 | 0.0 | 0.8 |
|  | MeOH_80 °C | 15 | 19.06 | 2.9 | 12.1 | 3.0 | 5.6 | 2.5 | 0.0 | 1.1 |

**Table S3 –** Chemical composition and related statistical information of untreated and pretreated substrates expressed as total extractives, total structural sugars, and total lignin. Pretreatment media: distilled water and a 50% (*v/v*) methanol (MeOH) solution catalysed by 0.1% (*w/v*) sulfuric acid. Pretreatment temperature: ambient temperature (T_amb_) and 80 °C.

| **Substrate** | **Pretreatment Condition** | **Total Extractives** (g/100 g TS) | | | **Total Structural Sugars ^a^** (g/100 g TS) | | | **Total Lignin ^b^** (g/100 g TS) | | |
| --- | --- | --- | --- | --- | --- | --- | --- | --- | --- | --- |
|  |  | **Average** | **St Dev** | **Statistical information ^c^** | **Average** | **St Dev** | **Statistical information ^c^** | **Average** | **St Dev** | **Statistical information ^c^** |
| Hazelnut Skin | Raw | 35.02 | 0.02 | a | 13.72 | 0.08 | b | 39.66 | 0.09 | a |
|  | H_2_O_T_amb_ | 34.01 | 0.00 | ab | 13.97 | 0.45 | b | 35.55 | 0.20 | b |
|  | H_2_O_80 °C | 32.31 | 0.48 | c | 17.11 | 0.47 | a | 38.52 | 0.39 | a |
|  | MeOH_T_amb_ | 33.75 | 0.22 | b | 14.53 | 0.55 | b | 36.22 | 0.60 | b |
|  | MeOH_80 °C | 30.42 | 0.21 | d | 17.08 | 0.05 | a | 36.42 | 0.37 | b |
| Almond Shell | Raw | 7.50 | 0.05 | a | 41.21 | 0.13 | c | 37.04 | 0.37 | c |
|  | H_2_O_T_amb_ | 3.80 | 0.05 | b | 40.64 | 0.30 | cd | 41.89 | 0.32 | a |
|  | H_2_O_80 °C | 6.48 | 0.54 | a | 43.44 | 0.19 | b | 39.02 | 0.15 | b |
|  | MeOH_T_amb_ | 3.71 | 0.24 | b | 44.54 | 0.30 | a | 41.05 | 0.68 | a |
|  | MeOH_80 °C | 3.99 | 0.34 | b | 40.28 | 0.12 | d | 41.80 | 0.21 | a |
| Spent Coffee Grounds | Raw | 28.98 | 0.52 | a | 43.20 | 0.07 | d | 18.71 | 0.36 | b |
|  | H_2_O_T_amb_ | 24.48 | 0.44 | c | 47.05 | 0.20 | b | 21.61 | 0.26 | a |
|  | H_2_O_80 °C | 26.18 | 0.23 | b | 43.54 | 0.34 | d | 20.88 | 0.38 | a |
|  | MeOH_T_amb_ | 23.57 | 0.04 | c | 48.61 | 0.10 | a | 21.07 | 0.26 | a |
|  | MeOH_80 °C | 24.61 | 0.07 | c | 45.96 | 0.04 | c | 20.51 | 0.00 | a |

*^a^ Total structural sugars are obtained as the sum of glucan, xylan, mannan, arabinan, galactan, and rhamnan.*

*^b^ Total lignin is calculated as the sum of acid soluble lignin and Klason lignin (Sluiter et al., 2008).*

*^c^ Not sharing letters means that the condition was significantly different (p < 0.05) than the compared condition.*
